# Reference Probe for TcpO_2_ at Rest: A Systematic Review

**DOI:** 10.3390/diagnostics13010077

**Published:** 2022-12-27

**Authors:** Judith Catella, Guillaume Mahé, Georges Leftheriotis, Anne Long

**Affiliations:** 1 Service de Médecine Interne et Vasculaire, Hôpital Edouard Herriot, Hospices Civils de Lyon, 69003 Lyon, France; 2 Laboratoire d’Excellence du Globule Rouge (Labex GR-Ex), Sorbonne, 75015 Paris, France; 3 Laboratoire Interuniversitaire de Biologie de la Motricité (LIBM) EA7424, Université Claude Bernard Lyon 1, Université de Lyon, 69008 Lyon, France; 4 UMR 5305: Laboratoire de Biologie Tissulaire et Ingénierie Thérapeutique, CNRS/Université Claude Bernard Lyon 1, Institut de Biologie et Chimie des Protéines, 69367 Lyon, France; 5 Unité de Médecine Vasculaire, CHU de Rennes, 35033 Rennes, France; 6 Inserm CIC 1414, Université de Rennes 1, CEDEX 9, 35033 Rennes, France; 7 Centre Hospitalier Universitaire de Nice, Unité de Médecine et Physiologie Vasculaire, Université Côte d’Azur, LP2M CNRS-7073, 06200 Nice, France

**Keywords:** peripheral arterial disease, TcpO_2_, reference probe, regional perfusion index

## Abstract

(1) Background: Transcutaneous oxygen pressure (TcpO_2_) is used to determine the severity of lower extremity arterial disease (LEAD). Many authors used a ratio of limb to chest TcpO_2_, also called the regional perfusion index (RPI), which should be independent of variations in oxygen delivery and reflective of local limb oxygen supply. The relevance of a reference probe-positioned TcpO_2_ electrode is debated. We aimed to review the relevance of the reference probe in previous studies using rest TcpO_2_. (2) Methods: We searched Medline and the Cochrane Central Register of Controlled Trials on 22 September 2022 using keywords related to TcpO_2_, reference probe and LEAD. (3) Results/Discussion: Fifteen studies were included in the review. Nine studies investigated LEAD severity (n = 9), amputation healing predication (n = 4), surgical outcome prediction (n = 2), therapeutic effect (n = 3) and difference according to diabetic status (n = 1). Four studies investigated more than 1 indication. Among 12 (16.7%) studies using RPI, only two authors found a benefit of using RPI instead of absolute TcpO_2_. Using only univariate analysis, one author reported that RPI was significantly related to viability at 1 year, while distal TcpO_2_ was not, on 13 limbs. The following year, the same author published a new study including 118 limbs that reported that RPI and absolute TcPO_2_ were both prognostic factors for limb viability at 1 year using a multivariate model. (4) Conclusions: Only one study firmly supporting the use of RPI, calculated using a reference probe on the arm, to predict BKA healing. Prospective studies are needed to validate this result; for other indications there is insufficient data supporting the use of a TcpO_2_ reference probe at rest.

## 1. Introduction

A chronic wound is one that fails to progress through a normal, orderly and timely sequence of repair, or in which the repair process fails to restore anatomic and functional integrity after three months [1]. In case of chronic wound, recommendations are to screen for lower extremity arterial disease (LEAD) using toe-brachial index [2]. However, systolic toe pressure (STP) cannot be measured in case of amputation or wound on the first toe.

Transcutaneous oxygen pressure (TcpO_2_) is a direct and non-invasive technique for skin oxygenation evaluation. Using a Clark electrode, PO_2_ is measured by a platinum cathode and a silver anode covered with a thin membrane that is permeable to oxygen [3]. The electronic reduction of oxygen allows a current to flow that is proportional to the partial pressure of oxygen. To ensure dermal oxygen permeability, the electrode is heated creating local hyperthermia, liquefying the crystalline structure of the stratum corneum and creating underlying capillary vasodilatation allowing more oxygen diffusion [4]. However, the TcpO_2_ reading is affected by numerous factors including the temperature of the tissues, shift of hemoglobin dissociation curve, degree of oxygen metabolism in tissues, circulatory status, peripheral blood perfusion, local skin, and anatomical conditions [5]. Initially used to study limb ischemia 40 years ago [6], it is currently used to determine the severity of LEAD [7]. TcpO_2_ is well correlated with the Fontaine classification for clinical severity, which varies from asymptomatic patients, to intermittent claudication, and finally chronic limb threatening ischemia (CLTI). An ideal perfusion assessment test for CLTI should have a good prognostic value to identify whether there is an adequate blood supply to the extremity for timely wound healing and to reduce both major and minor amputations [8]. In this regard, TcpO_2_ is well associated with the risk of amputation [7]; in addition, the value provided is a reflection of LEAD together with other dysfunctions, such as those related to diabetic microvascular, anemia, or abnormalities in hemoglobin oxygen-carrying capacity [9]. It has been shown that the TcpO_2_ values reflect variations of the total systemic oxygen delivery [10], which itself depends in part on the cardiac index [11]. Considering that the chest is a normally perfused location, many authors used a ratio of limb to chest TcpO_2_, also called regional perfusion index (RPI) or TcpO_2_ index, which should therefore be independent of variations in oxygen delivery and reflective of local limb oxygen supply [12,13,14,15]. Such a reference probe is useful during exercise tests because this affects the cardiopulmonary function [12]. However, for restinf TcpO_2_ measurement, in the absence of published guidelines on how to conduct it, the relevance of a reference probe-positioned TcpO_2_ electrode is debated; this is highlighted by the variability in practice. For instance, in a French national survey including 316 vascular physicians and surgeons, only 16.9% of the 213 responders used a chest reference probe at rest [16]; this is further supported by Leenstra et al., who reviewed 36 studies investigating TcpO_2_ in the assessment of CLTI and found that 16.7% used a reference probe [17]. It is of note that this review found that the reference probe was applied either on the chest (n = 4), the arm (n = 1) or the contralateral limb (n = 1) [17], but that only one study used reference probe values for the interpretation of TcpO_2_ [18].

We therefore aimed to review the relevance of the reference probe in previous studies using rest TcpO_2_, irrespective of indication, in order to identify the situations where this would be of value in order to adapt technical guidelines.

## 2. Materials and Methods

Studies eligible for this review were those that investigated the use of TcpO_2_ in patients with LEAD and that used a reference probe placed elsewhere than on the legs.

### 2.1. Search Strategy

We searched Medline and the Cochrane Central Register of Controlled Trials on 22 September 2022 using keywords related to TcpO_2_, reference probe, and LEAD. (“TcpO_2_” [All Fields] OR “transcutaneous oxygen pressure” [All Fields] OR “transcutaneous oxygen tension” [All Fields]) AND (“reference probe” [All Fields] OR “chest” [All Fields] OR “reference” [All Fields]) AND (“peripheral artery disease” [All Fields] OR “critical limb ischemia” [All Fields] OR “chronic limb threatening ischemia” [All Fields] OR “arterial claudication” [All Fields]) NOT “review” [Publication Type]. In addition, we screened records in the “Related citations in PubMed” section, and conducted a snowballing procedure to examine the references cited in systematic reviews retrieved through the systematic search. No time limit was applied to the searches. Language was restricted to English and French. Case reports were excluded, as were studies of exercise TcpO_2_.

### 2.2. Study Selection

J.C. reviewed and screened the title and abstract of potentially relevant records and determined final eligibility through examination of full texts. Each eligible study was screened searching for reference probe placement for at rest TcpO_2_ in prospective or retrospective studies.

### 2.3. Data Extraction

JC extracted information on number of participants, methodology used to apply probes, added value of reference probe and findings using reference probe.

The study was reported in accordance with the preferred reporting items for systematic reviews and meta-analyses (PRISMA) statement [19].

## 3. Results

**Figure 1:**
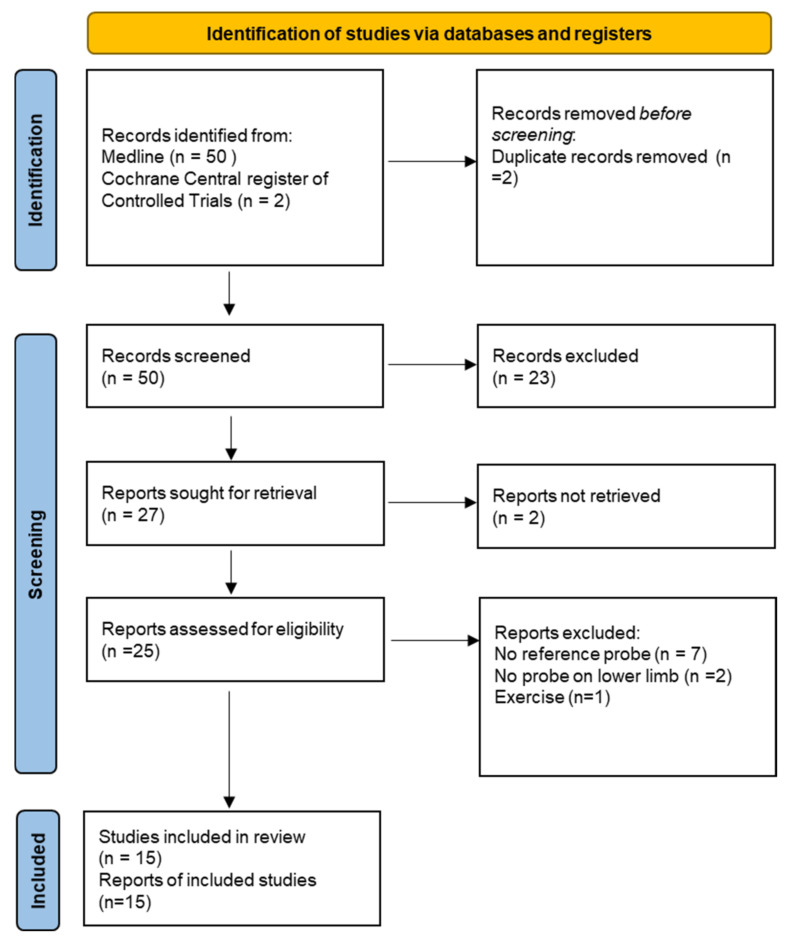
Flow chart.

The electronic search identified 52 records, two of which were duplicates. The title and abstract of 50 individual study records were assessed, 23 of which were irrelevant and therefore excluded. The remaining 27 records underwent full text examination; 15 studies were included in the review (Figure 1).

An overview of the study characteristics and results is presented in Table 1.

### 3.1. Indication of TcpO_2_ Measurement

Seven studies investigated LEAD severity [9,10,15,17,18,19,20], one study evaluated LEAD diagnosis [12], five studies investigated amputation healing prediction [13,15,20,21,22], and two investigated surgical outcome prediction [9,16]. Three studies investigated the effect of treatment; iloprost infusion [15], oxygen inhalation and praxilen infusion [20], and autologous transplantation of bone marrow mononuclear cells on CLTI [21]. One study evaluated differences in TcpO_2_ according to diabetic status [22]. Two studies investigated more than one indication [9,15].

### 3.2. Location of Reference Probe

For 14 studies (93.3%) the reference probe was on the chest (this was “on the chest in the subclavicular position” [7],”under the clavicula” [23], “5 cm under midclavicular” for two studies [8,22], and “5 to 7 cm infraclavicular, midaxillary line” [9], “on the chest wall over the second rib interspace, in the left midclavicular line” [20], “over the sternum” [16], “the anterior chest wall” [21], “on the anterior upper middle chest wall skin” [19] and on the chest without more details for the six other studies [10,15,17,18,24]); for the remaining study it was on the arm [13].

A total of 12 studies used RPI (the ratio between chest/arm and leg/foot TcpO_2_ [7,8,9,10,13,15,17,18,21,22,23,25]; the remaining three applied a reference probe without RPI calculation [19,20,24].

### 3.3. Relevance of Reference Probe

Dowd et al. [20] applied TcPO_2_ on the skin of the dorsum of the foot and on the skin of the chest wall over the second rib interspace, in the left midclavicular line in 91 normal volunteers. In the same study, they also measured TcPO_2_ on the foot in 66 patients with LEAD and in 24 patients undergoing amputations; however, they did not place a reference probe on the chest. A similar range of TcpO_2_ was found on the chest wall and on the dorsum of the foot. Authors did not calculate RPI [20].

The ischemic state of the lower extremities in 47 patients with LEAD (including eight acute ischemia and 59 chronic LEAD) was studied for the pretibial and dorsal skin of the leg [19]. The TcpO_2_ of the anterior upper middle chest wall skin was measured at rest as a standard value. The control study group was composed of 20 normal healthy males without respiratory or cardiovascular diseases. There was a significant correlation between the pretibial or chest wall TcpO_2_ and PaO_2_. The correlation of the pretibial TcpO_2_ with PaO_2_ was much closer (r = 0.71; *p* < 0.01) than that of the chest wall (r = 0.51; *p* < 0.05). There was a correlation between pretibial TcpO_2_ and aging (r = −0.48; *p* < 0.01); however, little correlation was noted between chest wall TcpO_2_ and aging in the normal subjects (r = −0.24; results not significant). In control normal males, mean TcpO_2_ at rest was 73 ± 11 mm Hg at the chest wall, 70 ± 9 mmHg at the pretibial skin, and 67 ± 10 mmHg at the dorsal skin of the leg. In chronic LEAD patients, mean chest TcpO_2_ at rest was 69 ± 18 mmHg and pretibial TcpO_2_ was 49 ± 19 mm Hg while in cases with acute ischemia, mean chest TcpO_2_ was 62 ± 14 mmHg and pretibial TcpO_2_ was 23 ± 30 mm Hg at rest. Authors do not calculate RPI. It is of note that, at rest, the mean pretibial TcpO_2_ decreased as the ischemia of legs became worse. That is, in both controls and grades of clinical severity (Grade 1, no symptoms; Grade 2, claudication; and Grades 3 and 4, rest pain and gangrene), the mean pretibial TcpO_2_ at rest decreased from 70 mmHg (control) to 63 mmHg (Grade 1), 56 mmHg (Grade 2), and 36 mmHg (Grades 3 and 4). There were statistically significant differences of the mean pretibial TcpO_2_ between control and every grade except Grade 1 [19].

Performing a retrospective matched paired study among 60 diabetic and 60 non-diabetic suspected CLTI patients, Biotteau et al. [24] observed that TcpO_2_ measured at the chest was lower in diabetic patients (mean, 53 mmHg) compared to non-diabetic patients (mean, 60 mmHg, *p* < 0.01). There was no significant difference between TcpO_2_ measured on the foot in diabetic patients (mean, 12 mmHg) and non-diabetic patients (mean, 15 mmHg). A multi-parametric step-by-step regression analysis showed that TcpO_2_ measured at the chest was inversely associated with weight, then with diabetes and gender [24].

Among 12 studies using RPI, only two (16.7%) reported a benefit of using RPI instead of absolute TcpO_2_ value [10,13]. Results are reported according to TcpO_2_ indication.

- LEAD diagnosis

Hauser et al. [7] studied 47 limbs in 24 patients, 12 of whom were young healthy subjects (25–35 years old) and 12 of whom had a clinical diagnosis of intermittent claudication. Supine thigh (57.3 ± 8.9 mmHg), calf (48.7 ± 9.3 mmHg), and foot (45.6 ± 12.4 mmHg) TcpO_2_ in claudicators were always significantly lower than those seen in young normal subjects (73.8 ± 10.9 mmHg, 70.0 ± 12.1 mmHg and 69.8 ± 5.3 mmHg, respectively). Despite the observed differences in chest and limb TcpO_2_, the RPI was strikingly similar in all subjects [7].

- LEAD severity

Cina et al. [15] studied 32 asymptomatic subjects and 85 LEAD patients in five groups: (1) patients with (2) claudication (n = 31), (3) rest pain (n = 26), (4) impending gangrene (n = 12), or (5) ischemic ulcers (n = 16) [15]. Mean TcpO_2_ at the chest was the same in all groups (65 mmHg). At the calf and foot, statistically significant differences were found between normal subjects and claudicants, between claudicants and patients with rest pain, and between those with rest pain and impending gangrene (*p* < 0.001, 0.002, and 0.03, respectively, at the calf and *p* < 0.0001 in all cases at the foot). They found that RPI did not improve its ability to determine severity (claudication, rest pain, or impending gangrene) [15].

Lalka et al., included 62 revascularized patients (89 limbs) in their studies. Thirty five limbs were only associated with claudication and 54 limbs were considered to have CLTI defined as rest pain, dependent rubor, ischemic ulcers, or gangrene. Patients with claudication and CLTI had significant different TcPO_2_ value (*p* = 0.003). Only 40 of 89 limbs had RPI calculation and there were also a significant difference of RPI between patients with claudication and CLTI (*p* = 0.013). Absolute TcpO_2_ value and RPI were relatively poor for determination of a limb threat threshold to separate patients with claudication and patients with CLTI. False-negative error rate for RPI was rate less than 20% (i.e., 16.7 ± 15.2%); however, the large standard deviation and high false-positive error rate (26.5 ± 7.6%) caused this measurement to be no more adequate than absolute TcpO_2_ [9].

Similarly, Moosa et al., found that absolute TcpO_2_ and RPI were equally effective in monitoring LEAD in another 22 patients with claudication or rest pain [17].

Evaluating the effect of 28 days of iloprost and retraining on 13 limbs’ preservation in 12 patients with LEAD at Leriche-Fontaine Stage III or IV, Chomard et al., found that median values of RPI measured in the vertical position were related to viability (*p* = 0.016), while distal TcpO_2_ on the foot (supine or vertical) was not (*p* = 0.194) [10]. The decision to amputate was based on clinical criteria (pain, trophic appearance, risk of infection). Treatment with iloprost saved the limb in 53.85% of cases (n = 7/13) at 1 year in patients in whom amputation had been deemed initially necessary [10].

Chomard et al. [18] sought to identify and define objective prognostic criteria of viability at 1 year of a limb with CLTI. The study involved 116 patients with Leriche and Fontaine stage III or IV LEAD. A multivariate logistic regression found six factors prognostic of limb viability at 1 year with threshold values: age (<75 years), TcpO_2_ absolute value after verticalization (>41 mmHg), TcpO_2_ after 1 minute’s inhalation of oxygen (<45 mmHg), TcpO_2_ after 4 minutes’ inhalation of oxygen (>62 mmHg), slope of TcpO_2_ between 1 and 4 minutes’ oxygen inhalation (<8.06), slope of RPI between 1 and 4 minutes’ oxygen inhalation (>−0.33) [18].

- Determination of clinical revascularization success

After revascularization, Osmundson et al., observed that RPI and absolute TcpO_2_ value increased substantially [16]. Lalka et al., compared postoperative TcPO_2_ according to clinical evaluation before discharge: revascularization was a success when symptoms and signs of limb ischemia significantly improved, while failure corresponded to no change or worsening of ischemic signs and symptoms [9]. TcPO_2_ and RPI were both significantly associated with clinical success (*p* < 0.001) (9).

- Prediction of amputation healing

Evaluating 35 patients with LEAD requiring amputation, significant correlations were found between absolute TcpO_2_ and RPI at the foot and below-knee sites [21]. In Cina et al. [15], 22 amputations were performed without revascularization. Selection of the amputation level was made on the basis of clinical judgment and was not influenced by the measured values of TcpO_2_. In 17 cases with a successful outcome, the preoperative TCpO_2_ value at the level of amputation ranged from 38 to 62 mm Hg. Five amputations did not heal, with pre-amputation TcpO_2_ values ranging from 2 to 42 mm Hg. The difference in TCpO_2_ between patients with good and poor results was statistically significant (*p* < 0.001). Use of the RPI, however, did not improve the discrimination between patient groups [15].

In 1983, Mustapha et al., found a critical value of approximately 35 mmHg to predict successful amputation. Determination of the 10 cm below knee-control site ratio did not appear to provide any further advantage over the absolute 10 cm below knee site value in predicting amputation success [8].

Kram et al., used a brachial reference probe and the calf for TcpO_2_; RPI improved the performance of TcpO_2_ in predicting wound healing after below the knee amputation (BKA) in a subpopulation with low TcpO_2_; calf TcpO_2_ > 20 mmHg predicted successful healing after BKA with a sensitivity of 82%, specificity of 86%, and overall accuracy of 83%, and among those with a TcpO_2_ < 20 mmHg the RPI > 0.20 predicted successful healing after BKA with a sensitivity of 100%, specificity of 86% and overall accuracy of 98% [13]. They found no advantage in using the RPI threshold in patients with calf TcpO_2_ > 20 mmHg (most of such patients [26,27] had successful healing after BKA) [13].

- Estimatiin of medical treatment effect

In a group of 20 patients with LEAD, including 23 limbs, Mustapha et al. [22] evaluated the effect of oxygen inhalation and praxilen infusion on TcpO_2_ absolute value and RPI. Absolute TcPO_2_, measured 10 cm below the knee, was significantly higher after praxilene infusion (mean ± SD: 42.05 ± 14.83 vs. 51.74 ± 17.26, *p* < 0.001), oxygen inhalation (42.05 ± 14.83 vs. 56.77 ± 18.49, *p* < 0.001) and concomitant praxilene and oxygen treatment (42.05 ± 14.83 vs. 64.13 ± 19.80, *p* < 0.001), while RPI increased significantly only after post praxilene infusion (0.68 ± 0.22 vs. 0.86 ± 0.26, *p* < 0.001) [22].

De Vriese et al. [23] enrolled 16 patients with CLTI (including ischemic rest pain, minor tissue loss and major tissue loss), present for a minimum of 2 months without evidence of improvement in response to medical treatment, including wound care, drug therapy and an exercise training program. Mononuclear cells were injected into the gastrocnemius muscle of the ischemic leg. The primary end-points were healing the most important lesion whilst avoiding amputation and the relief of rest pain from baseline to 12 weeks of follow-up. The changes in TcpO_2_ were a secondary end-point. Sixteen patients were treated with mononuclear cells. Eight patients were not evaluable: four deaths, three amputations and one revascularization. RPI was 0.51 ± 0.11 at baseline, 0.49 ± 0.11 at 6 weeks (*p* = 0.79 vs. baseline) and 0.86 ± 0.03 at 12 weeks (*p* < 0.001 vs. baseline). Absolute TcpO_2_ values were not reported in this study [23].

## 4. Discussion

Among 15 studies included in the review, only two studies found a benefit in using RPI instead of absolute TcpO_2_ [15,18].

CLTI is a clinical syndrome defined by the presence of a LEAD in combination with rest pain, gangrene, or a lower limb ulceration lasting >2 weeks as a result of arterial insufficiency (deficient or absent blood supply) [7]. Usually, the impairment of peripheral perfusion is a chronic process that occurs over months or years, as well as with cardiovascular risk factors such as smoking, diabetes, hypertension, dyslipidemia, and chronic kidney disease [7]. The benefit of TcpO_2_ for the diagnosis of CLTI and evaluation of therapy in patients with CLTI is strongly debated. Reliability is limited by the difficulties related to the strict exam conditions; patients should avoid smoking and caffeine intake before the test and it needs to be performed in a temperature-controlled environment. Studies have shown poor-to-moderate reliability and reproducibility [23], with a sensitivity ranging from 0.61 to 0.81 for the prognosis of diabetic foot ulcer healing. Compared to systolic toe pressure, one study found that TcpO_2_ was a better predictor for ulcer healing than STP in diabetic patients with chronic foot ulcers [24]; more recently, however, using the COPART registry STP < 30 mmHg was found to be better than TcpO_2_ < 10 mmHg in predicting amputation with an area under the curve of the receiver operating curve of 0.678 for STP versus 0.638 for TcpO_2_ [25]. Nonetheless, TcpO_2_ < 10 mmHg predicted major amputation (odds ratio, OR: 2.3, 95% confidence interval, CI [1.5; 3.5]) as well as TcPO_2_ < 30 mmHg (OR: 3, 95%CI [1.3; 6.9]) [25].

In a preliminary study that included 12 patients (13 limbs), Chomard et al. [15] reported that RPI measured in a vertical position was significantly related to viability at 1 year while distal TcpO_2_ on the foot was not. However, confounding bias cannot be excluded as the authors did not investigate this using multivariate analysis and it is of note that only very few patients/limbs were included. The following year, Chomard et al. [17] published a new study including 116 patients (118 limbs) that reported that RPI and absolute TcPO_2_ were both prognostic factors for limb viability at 1 year using a multivariate model. The other study that found that RPI was better than absolute TcpO_2_ was reported by Kram et al. [18] who used a method to predict wound healing after BKA that, to the best of our knowledge, was not subsequently used in any published study. It is of note that the reference probe was placed on the middle of the arm, which, as the authors suggest, may increase reproducibility, but the calculation of the RPI using the lower TcpO_2_ value, measured on either the anterior or posterior calf, is questionable. In addition, the added value of RPI was evaluated only among those with calf TcpO_2_ ≤ 20 mmHg (n = 12); more than two-thirds of patients had a calf TcpO_2_ > 20 mmHg and all-but-one of these healed.

RPI takes into consideration intra-individual changes in blood oxygenation and therefore would be particularly interesting in patients who have cardiac or pulmonary diseases that produce variable degrees of oxygen desaturation (e.g., respiratory insufficiency or chronic cardiac failure). However, we did not find any study reporting TcpO_2_ values in patients with central circulatory failure. It is of note that Stosseck et al., reported that large changes of blood pressure did not influence the TcpO_2_ until the systolic pressure fell <90 mmHg, and that a further 10-mmHg decrease of systolic pressure caused an additional 10% underestimation of TcpO_2_ [28]. As blood pressure and SpO_2_ may be easily measured during TcpO_2_ recording to eliminate respiratory insufficiency or central circulatory failure, this suggests that RPI is not of particular importance. Furthermore, if a patient has a central circulatory failure demonstrated by a low TcpO_2_ reference probe value (or altered RPI), this would also worsen healing prognosis for the patient and should be taken into account.

A potentially important point is the lack of precision in the exact location of the reference probe on the chest in the majority of studies that used this site [14,15,22,26,27,29,30,31,32]. However, Orenstein et al., investigated the variability of TcpO_2_ measurements over 23 different locations in healthy volunteers (including above and below the nipple) [33]. The mean TcpO_2_ for all measurement was 61 ± 8 mmHg; the lowest values (less than 90% of the mean) were recorded on the forehead, cheek and medial aspect of the leg, and the highest values (more than 110% of the mean) on the inferior scapula range, chest (below the nipple) and iliac crest [33]. There was little difference between above (103% of the mean) and below the nipple (110% of the mean), which suggests little variation, but this study does not exclude greater variation between other areas of the chest.

Another consideration is that TcpO_2_ could function in increasing age because of the thickening of the skin and the reduction of capillary density [34,35]. This was reported by Hauser et al., who found a statistically significant lower mean chest TcpO_2_ value in those aged 55–65 years compared to those aged 25–35 years [12], and Cina et al., described a linear relation between decreasing TcpO_2_ with increasing age [26]. RPI should not be affected by age, and this could therefore be pertinent. However, Chomard et al., found there was no advantage in using RPI rather than TcpO_2_ as an absolute value to predict limb viability at 1 year [31].

In this systematic review, limitations are mostly due to the literature search, which was limited to articles published in either French or English language, and two reports of studies were not retrieved. Another limitation is heterogeneity of the included studies, with multiple different outcomes of interest and different populations, reflecting past researches.

## 5. Conclusions

Existing literature does not support routine use of reference probe. We found only one study firmly supporting the use of RPI calculated using a reference probe. It is of note that the latter was placed on the arm and the indication was to predict BKA healing. Further prospective studies are needed to validate this result but for other indications there is insufficient data supporting the use of a TcpO_2_ reference probe at rest.

## Figures and Tables

**Table 1:** Overview of included studies.

Reference	Participants	Mean Age ± SD (Years)	Probe Placement	TcpO_2_ Indication	Control Site Value	Findings
Control site with RPI calculation						
Hauser et al. [5]	12	61 ± 8	Subclavicular, thigh, calf, and dorsum of the foot	LEAD severity	-	Despite the observed differences in chest and limb TcpO_2_, the RPI is strikingly similar in all subjects.
Mustapha et al. [6]	NA	NA	5 cm below the midclavicular line; 10 cm below-knee	LEAD severity; amputation healing prediction	-	No further advantage in determining the RPI to predict amputation success.
Cina et al. [13]	22 + 100	NA	Calf, dorsum of the foot, thigh, and in proximity to any lesions; chest	LEAD severity	-	RPI was equally effective in identifying the presence of LE-PAD and accurately characterized different degrees of severity (claudication vs. rest pain vs. impending gangrene; *p* < 0.001).
Mustapha et al. [20]	20	NA	5 cm below the midclavicular line; 10 cm below-knee	Therapeutic effect evaluation	-	Oxygen inhalation increases significantly the TcpO_2_ absolute value while Praxilene infusion does not. Conversely, praxilene infusion increases RPI while oxygen inhalation does not.
Osmundson et al. [14]	20	NA	Foot and chest	LEAD severity	-	No advantage to adding RPI to estimate probability of clinical improvement (pain or healing) after revascularization.
Lalka et al. [7]	62	65	Foot and chest	LEAD severity and surgical outcome prediction	-	Before surgery, TcpO_2_ ≤ 22 mmHg and RPI ≤ 0.46 indicate severe limb ischemia requiring urgent revascularization. After surgery, TcpO_2_ ≤ 22 mmHg and RPI ≤ 0.53 indicate that revascularization is likely to fail.
Moosa et al. [15]	22	NA	Foot and chest	LEAD severity and surgical outcome prediction	-	RPI is equally effective in monitoring LE-PAD.
Kram et al. [11]	40	50 ± 18	Calf and brachial; RPI = the lesser of the anterior and posterior calf/brachial TcpO_2_	Amputation healing prediction	+	The use of RPI > 0.20 as predictive of successful healing after BKA was associated with a sensitivity, specificity, and overall accuracy of 100%, 86% and 98%, respectively, compared to 82%, 86% and 83%, respectively, by use of absolute calf TcpO_2_ values.
Mars et al. [19]	35	NA	Chest, above-knee, below-knee and mid-foot	Amputation healing prediction	-	RPI and absolute TcpO_2_ are good predictors of the potential of an amputation site to heal.
Chomard et al. [8]	12	71	Foot ant Chest	Therapeutic effect evaluation	+	Supine RPI and vertical RPI, with values of 36.67 and 65.08, respectively, appeared to be significantly linked to the preservation of limb.
Chomard et al. [16]	116	Men: 71.9 ± 12.8; Women: 81.6 ± 9.3	Foot and chest	LEAD severity	-	6 factors prognostic of limb viability at 1 year with threshold values: Age (70–75), TcpO_2_ after verticalization (41 mmHg), TcpO_2_ after 1 minute’s inhalation of oxygen (45 mmHg), TcpO_2_ after 4 minutes’ inhalation of oxygen (62 mmHg), Slope of TcpO_2_ between 1 and 4 minutes’ oxygen inhalation (8.06), Slope of RPI between 1 and 4 minutes’ oxygen inhalation (−0.33).
De Vriese et al. [21]	16	78.2	On the dorsalis pedis artery and under the clavicula	Therapeutic effect evaluation	NA	Effect of autologous transplantation of bone marrow mononuclear cells on CLTI. No comparison between RPI and absolute value. RPI increase after treatment in 8 patient which did not need amputation.
Control site without RPI calculation						
Dowd et al. [18]	161 + 62	66	Foot; above and below the knee; 2nd rib midclavicular lign	LEAD severity; amputation healing prediction	NA	Mean ± SD TcpO_2_ values at the pretibial site (70 ± 9 mmHg) and chest wall (73 ± 11 mmHg) were close.
Ohgi et al. [17]	20 + 47	57	Pretibial and dorsal skin; Chest	LEAD severity	NA	Closer correlation of the pretibial TcPO_2_ with PaO_2_ (r = 0.71; *p* < 0.001) than that of the chest wall (r = 0.51; *p* < 0.05).
Biotteau et al. [22]	60 + 60	NA	Foot and chest	Diabetic status evaluation	NA	Chest-TcpO_2_ were higher in non-diabetic patients while there were no difference at foot level.

Abbreviations: LEAD, Lower Extremity Arterial Disease; RPI, Regional Perfusion Index; TcpO_2_, Transcutaneous Pressure of Oxygen; CLTI, Critical Limb of Threatening Ischemia; BKA, Below-the-Knee Amputation; NA, Not Applicable; SD, Standard Deviation; Control site value: +, reference probe allow to improve TcPO_2_ accuracy; -, reference probe do not allow to improve TcPO_2_ accuracy; NA, insufficient data to conclude on reference probe value.

## Data Availability

Not applicable.
